# SGLT2 Inhibitors in the Management of Cardio-Renal-Metabolic Syndrome: A New Therapeutic Era

**DOI:** 10.3390/medicina61111903

**Published:** 2025-10-23

**Authors:** Konstantinos Grigoriou, Paschalis Karakasis, Athina Nasoufidou, Panagiotis Stachteas, Aleksandra Klisic, Efstratios Karagiannidis, Barbara Fyntanidou, Djordje S. Popovic, Dimitrios Patoulias, Antonios P. Antoniadis, Nikolaos Fragakis

**Affiliations:** 1 Department of Pharmacology, University of Athens, 75 Mikras Asias Avenue, Goudi, 11527 Athens, Greece; dinosgrigoriou@gmail.com; 2 Second Department of Cardiology, Hippokration General Hospital, Aristotle University of Thessaloniki, Konstantinoupoleos 49, 54642 Thessaloniki, Greece; athinanassi@gmail.com (A.N.); aantoniadis@gmail.com (A.P.A.); fragakis.nikos@googlemail.com (N.F.); 3 Faculty of Medicine, University of Montenegro, 81000 Podgorica, Montenegro; aleksandranklisic@gmail.com; 4 Emergency Department, AHEPA University General Hospital, Aristotle University of Thessaloniki, 54636 Thessaloniki, Greece; stratoskarag@gmail.com (E.K.); fyntanidou@auth.gr (B.F.); 5 Clinic for Endocrinology, Diabetes and Metabolic Disorders, Clinical Centre of Vojvodina, Medical Faculty, University of Novi Sad, 21000 Novi Sad, Serbia; pitstop021@gmail.com; 6 Second Propedeutic Department of Internal Medicine, Faculty of Medicine, School of Health Sciences, Aristotle University of Thessaloniki, 54642 Thessaloniki, Greece; dipatoulias@gmail.com

**Keywords:** cardio-renal-metabolic syndrome, cardiorenal syndrome, SGLT2 inhibitors, chronic kidney disease, heart failure, diabetes, obesity, metabolic disorders

## Abstract

Cardiovascular (CV) disease, chronic kidney disease, obesity, and diabetes mellitus have reached epidemic proportions over the past few decades. Accumulating evidence highlights the strong interconnection between these conditions, leading to the definition of a broader disease entity known as cardio-renal-metabolic (CRM) syndrome. This newly recognized clinical entity presents important challenges in identifying the optimal treatment strategy within a holistic, patient-centered framework. In line with this, sodium glucose cotransporter 2 inhibitors (SGLT2is), owing to their multifaceted pharmacological effects, have been suggested as possible treatment options in the management of CRM. SGLT2is exert their antihyperglycemic effects by impeding the renal reabsorption of sodium and glucose, causing glycosuria and natriuresis. Research has confirmed that their unique beneficial effects extend beyond glycemic control, reducing CV death and hospitalizations in patients with heart failure, and the incidence of kidney failure in dedicated kidney outcome studies—regardless of diabetes status. Furthermore, these agents contribute to weight loss and blood pressure reduction. Their benefits appear to stem from a combination of factors, which include reduced oxidative stress, lower levels of inflammation, regulated neurohormonal activation, improved endothelial function, and enhanced metabolic efficiency. This review aims to provide a comprehensive analysis of the pathophysiological mechanisms underlying the effects of SGLT2is in CRM syndrome, synthesize evidence from landmark clinical trials, evaluate current experimental and diagnostic approaches, and provide the emerging role of SGLT2is in the treatment of this new clinical entity.

## 1. Introduction

Over the past few decades, obesity and diabetes mellitus (DM) have emerged as major global health epidemics, with their prevalence rising sharply due to widespread changes in lifestyle, diet, and environmental factors [1,2,3,4]. At the same time, cardiovascular disease (CVD) and chronic kidney disease (CKD) have remained persistent public health concerns, contributing substantially to global morbidity and mortality. CVD continues to be the leading cause of mortality worldwide, while CKD has seen a steady increase in both incidence and progression, often remaining underdiagnosed until advanced stages [5,6].

Although traditionally viewed as separate disease entities, these conditions often coexist in clinical practice and have a significant overlap in their underlying pathophysiological pathways, such as inflammation, oxidative stress, and neurohormonal activation [7,8]. To emphasize this complex interplay, recently the American Heart Association (AHA) introduced the cardiovascular–kidney–metabolic (CKM) syndrome, an equivalent term for CRM syndrome, as a conceptual framework that reflects the multidirectional and synergistic relationships among the heart, blood vessels, kidneys, and metabolism [9]. Recognizing CRM syndrome is essential for advancing more integrated approaches to prevention, diagnosis, and treatment. The CRM syndrome is divided into five stages: stage 0, no CRM risk factors; stage 1, excess and/or disordered adiposity; stage 2, presence of metabolic risk factors (DM, metabolic syndrome, hypertension, dyslipidemia) or CKD with mild-moderate decrease in estimated glomerular filtration rate (eGFR); stage 3, subclinical CVD, CKD with moderate-severe decrease in eGFR; and stage 4, clinical CVD, heart failure (HF), and/or CKD with severe decrease in eGFR [9].

In the United States, CRM stage 0 was found in 17.35% of individuals aged 20–44, 5.45% of those aged 45–64, and only 1.80% of those aged 65 and older. As age increased, so did the prevalence of subclinical CRM syndrome (stages 1–3), with 80.94%, 85.95%, and 72.03% of individuals in these respective age groups affected. These trends highlight the rising burden of CRM risk factors with age, emphasizing the need for early prevention efforts [10].

**Figure 1:**
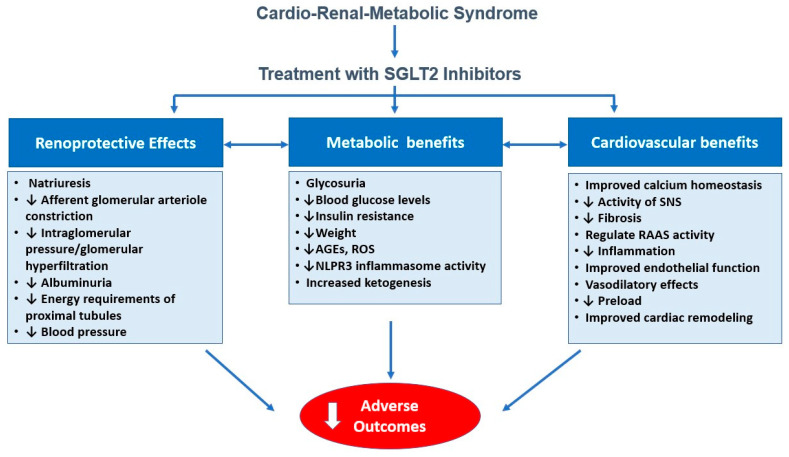
SGLT2is exhibit consistent benefits beyond glycemic control, including renoprotective and cardioprotective effects, and improvements in key components of metabolic syndrome such as hypertension and obesity. These effects are mediated through multiple pathophysiological pathways, including reductions in inflammation and oxidative stress, and improvements in endothelial function. Collectively, these mechanisms support the growing therapeutic role of SGLT2is in the comprehensive management of CRM syndrome. SGLT2is, sodium-glucose cotransporter 2 inhibitors; AGEs, advanced glycation end products; ROS, reactive oxygen species; NLPR3, NOD-like receptor protein 3; SNS, sympathetic nervous system; RAAS, renin–angiotensin–aldosterone system.

Due to the intricate interplay among metabolic dysregulation, endothelial dysfunction, oxidative stress, and inflammation that underpins CRM syndrome progression, therapeutic strategies targeting these detrimental pathways are increasingly necessary [9]. Typically, research and clinical practice have managed conditions such as HF, DM, and CKD in isolation, often overlooking their bidirectional interactions, which hinders the development of new drugs and therapeutic strategies. In this regard, sodium-glucose cotransporter 2 inhibitors (SGLT2is) have been recognized as promising therapeutic options due to their ability to modulate multiple pathophysiological aspects of this syndrome [10,11,12,13]. Originally created to lower blood glucose, SGLT2is have since shown notable protective effects on the heart and kidneys in a variety of clinical scenarios and patient groups, including individuals without DM [14,15,16]. Furthermore, accumulating evidence underscores the antihypertensive effects, weight loss benefits, and improvements in endothelial function associated with SGLT2is [16]. These findings suggest that these agents might deliver a novel disease-modifying strategy that complements conventional management. Nevertheless, future trials are warranted to define their optimal use and efficacy across the different stages of CRM syndrome. (Figure 1)

This review summarizes the current research on how SGLT2is affect CRM syndrome and explores the potential benefits of using them to better address the multifaceted clinical demands of patients with this condition.

## 2. Pathophysiology of Cardio-Renal-Metabolic Disease

An emerging body of experimental and clinical evidence underscores the complex pathophysiological connections between cardiovascular (CV), renal, and metabolic diseases. The three conditions are interrelated through common pathways, involving chronic inflammation, oxidative stress, neurohormonal dysregulation, aberrant secretion of chemical mediators, and fibrosis. These overlapping processes create a self-perpetuating cycle that accelerates CRM syndrome progression and increases the likelihood of adverse clinical outcomes [8,17,18].

Excessive and/or dysfunctional adiposity lies at the core of this syndrome and serves as the primary trigger for systemic inflammation and metabolic imbalance. Adipose tissue secretes several proinflammatory cytokines and adipokines, such as tumor necrosis factor-α (TNF-α), interleukins IL-6 and IL-1β, and leptin and resistin. These mediators cause chronic inflammation and oxidative stress, promote insulin resistance, enhance weight gain, and cause endothelial injury. All these processes subsequently contribute to the development of atherosclerotic cardiovascular disease (ASCVD) and CKD [17,19]. Adipose tissue also releases free fatty acids (FFAs), which further worsen insulin resistance by impairing insulin signaling and mitochondrial function. Additionally, FFAs disrupt lipid profiles by lowering high-density lipoprotein (HDL) levels and promoting the generation of small, dense low-density lipoprotein (LDL) particles, which are more atherogenic [20,21].

Insulin resistance emerges early and serves as a central node linking metabolic, CV, and renal dysfunction. It impairs glucose uptake, promotes low-grade inflammation and dyslipidemia, enhances sympathetic tone, and activates the renin–angiotensin–aldosterone system (RAAS), which results in impairment of cardiac and renal function [22,23]. Progression of insulin resistance can lead to T2DM. Chronic hyperglycemia further drives endothelial dysfunction and promotes the production of advanced glycation end-products (AGEs), which trigger inflammatory and fibrotic signaling pathways. These processes contribute to extracellular matrix (ECM) deposition, nephron loss, and a gradual decrease in glomerular filtration rate (GFR), while also accelerating plaque formation and cardiac fibrosis [24,25].

DM and insulin resistance often lead to elevated blood pressure. The underlying mechanisms include activation of RAAS and sympathetic nervous system (SNS), endothelial and mitochondrial dysfunction, increased activation of renal and endothelial sodium channels, inflammation, oxidative stress, vascular stiffness, volume expansion, abnormal gut microbiota, and enhanced renal SGLT2 activity [26,27]. The coexistence of hypertension and DM has detrimental effects on the heart and kidneys. In the kidneys, both conditions promote glomerular hyperfiltration during the initial phase, which can progress to glomerulosclerosis. Abnormal RAAS activation exerts multifactorial pathogenic renal effects, including increased intraglomerular pressure, proteinuria, fibrosis, and morphological changes in renal vasculature—such as afferent arteriolar hyalinosis and hyperplastic arteriosclerosis [25,28]. In CV system, hypertension and DM are major risk factors for macrovascular complications, including CVD and stroke, and are associated with higher CV mortality [29].

Obesity is a significant contributor to the development and progression of CV and kidney diseases. It partly increases this risk by being closely associated with DM and hypertension. But obesity also serves as an independent driver of these conditions [30]. The underlying mechanisms are multifactorial and interrelated, involving metabolic, hormonal, and hemodynamic changes. Key pathophysiological pathways include insulin resistance, chronic inflammation, heightened SNS activity, increased leptin production, and activation of the RAAS [30,31]. These factors promote structural and functional cardiac changes. Obesity often induces a high-output cardiac state, which can lead to eccentric left ventricular (LV) hypertrophy, enlargement of the left atrium, and impaired LV diastolic function. Over time, these changes cause ventricular dysfunction and HF [31,32].

In the kidneys, obesity promotes the development and progression of CKD through mechanisms such as glomerulosclerosis, tubular inflammation, and tubule-interstitial fibrosis [33]. Additionally, recent studies highlight the role of the adipose–brain–kidney axis in obesity-related kidney dysfunction. This axis involves the activation of sensory neurons in adipose tissue by local factors, which then signal brain centers that increase renal sympathetic nerve activity—exacerbating renal damage [34].

HF and CKD represent the unfortunate end-stage outcomes of CRM syndrome. The bidirectional relationship between these two conditions creates a vicious cycle in which declining heart function contributes to kidney deterioration, and vice versa. Various hemodynamic and neurohormonal abnormalities contribute to this reciprocal damage and perpetuate disease progression [35]. HF is associated with decreased cardiac output, effective hypovolemia, and peripheral vasoconstriction, all of which lead to inadequate renal perfusion, thereby promoting renal injury and atrophy [36]. On the other hand, CKD-associated activation of RAAS exacerbates hypertension and increases cardiac afterload and preload. These hemodynamic changes, together with uremic toxins, chronic inflammation, and oxidative stress, contribute to cardiac fibrosis and ventricular remodeling, leading to further deterioration of cardiac function—completing the vicious cycle [35].

In summary, CRM syndrome represents a convergence of metabolic, CV, and renal dysfunctions, initiated by adipose tissue–driven inflammation and propagated through neurohormonal and hemodynamic abnormalities. Recognizing this complex, bidirectional network of pathology underscores the importance of early, integrated, and multi-organ-targeted interventions to prevent or reverse disease progression.

## 3. Mechanisms of Action of SGLT2 Inhibitors Beyond Glycemic Control

SGLT2is represent a relatively recent class of oral antidiabetic agents that function by lowering the renal threshold for glucose reabsorption within the proximal convoluted tubules. This leads to inhibition of glucose and sodium reabsorption, resulting in glycosuria and natriuresis [1]. These medications have shown remarkable cardio–renal–metabolic benefits, underscoring their expanding therapeutic applications beyond glucose regulation and, as a result, have emerged as promising treatments for CRM syndrome [15,37,38,39].

### 3.1. Hemodynamic Effect: Natriuresis, Osmotic Diuresis, Blood Pressure Reduction

SGLT2is exert significant hemodynamic effects through mechanisms such as natriuresis and osmotic diuresis, which together lower blood pressure [40,41]. Because most sodium reabsorption takes place in the loop of Henle and distal tubule, the diuretic effect of SGLT2is alone is limited. However, in patients with HF, these agents can enhance the diuretic response when combined with other diuretics by improving sensitivity to atrial natriuretic peptide (ANP) [42]. The enhanced excretion of sodium further facilitates the removal of excess body fluid, thereby reducing extracellular fluid volume and ultimately decreasing the preload on the heart [43,44].

SGLT2is have been shown to modestly reduce blood pressure [45,46]. Beyond natriuresis and osmotic diuresis, it has been suggested that SGLT2is affect the RAAS by increasing sodium delivery to the nephrons; however, current evidence is inconsistent and does not uniformly support this hypothesis [45,46,47,48,49]. This class may exert additional antihypertensive effects through other mechanisms, including reduced arterial stiffness and vascular resistance (via unclear pathways), and suppression of SNS activity [50,51]. An additional factor that contributes to the antihypertensive effect of gliflozins is the weight reduction they promote, caused by glucose-related caloric loss [52]. Meta-analyses indicate that SGLT2is lead to a dose-dependent body weight reduction of about 1.5 to 2 kg relative to placebo [53,54,55].

### 3.2. Metabolic Modulation: Improved Mitochondrial Function and Substrate Utilization

Mitochondrial dysfunction is a central facet of many CV disorders and contributes to the development and progression of the main pathologies within the cardio-renal axis [56]. Both the myocardium and kidneys have high mitochondrial content due to their substantial energy demands, which makes them particularly susceptible to mitochondrial impairment [56]. Experimental animal studies have shown that SGLT2is reduce ROS production, decrease mitochondrial fragmentation, balance mitochondrial fusion and fission, enhance mitochondrial respiratory function, stimulate mitochondrial biogenesis, and regulate intracellular calcium handling, collectively contributing to normal cellular processes and the reversal of CRM syndrome [56,57,58].

Furthermore, SGLT2is promote a more favorable metabolic profile by enhancing substrate flexibility [59]. Beyond improving the utilization of ketone bodies, free fatty acids, and amino acids, reducing cytosolic sodium, and increasing ATP production, emerging evidence suggests that these agents exert broader effects on cellular energetics [60,61,62]. SGLT2is seem to trigger a starvation-like condition that activates nutrient-sensing pathways conserved through evolution, such as AMP-activated protein kinase (AMPK), sirtuins (SIRT1), and peroxisome proliferator-activated receptor gamma coactivator 1-alpha (PGC-1α), while simultaneously inhibiting anabolic signaling through suppression of the mechanistic target of rapamycin (mTOR) pathway [63,64,65,66].

This molecular response initiates a stimulation of autophagy, a critical cellular process responsible for clearing dysfunctional organelles, reducing oxidative stress, and maintaining mitochondrial function [67]. Enhanced mitophagy and the renewal of mitochondrial biogenesis have been consistently observed in animal models and cell cultures, including in tissues lacking SGLT2 expression, such as myocardium. The evidence indicates that SGLT2is might provide their cardioprotective and renoprotective effects by directly targeting nutrient and redox signaling pathways, independently of glycosuria or broader systemic metabolic alterations [58].

### 3.3. Anti-Inflammatory and Antifibrotic Pathways

Inflammation and oxidative stress are pivotal in the pathogenesis of CRM syndrome [67]. The anti-inflammatory effects of SGLT2is are well established [68]. These agents suppress the release of circulating pro-inflammatory and inflammatory cytokines such as C-reactive protein (CRP), TNF-α, IL-6, monocyte chemoattractant protein 1 (MCP-1), transforming growth factor-beta (TGF-β), ferritin, and leptin. They also modulate the activity of NOD-like receptor protein 3 (NLRP-3) inflammasome, leading to decreased secretion of IL-1β and IL-18 [68,69,70,71].

Moreover, SGLT2is exert antifibrotic effects through multiple mechanisms. As previously discussed, these agents help mitigate oxidative stress by enhancing mitochondrial function and limiting the production of reactive oxygen species (ROS) [72,73], which in turn attenuates pro-fibrotic signaling cascades, including the TGF-β/SMAD pathway and nuclear factor kappa B (NF-κb) [74]. Additionally, SGLT2is lower circulating levels of cytokines such as IL-6 and TNF-α, both of which contribute to cardiac fibroblast activation and extracellular matrix (ECM) remodeling [71,75,76]. Enhanced myocardial energy metabolism, facilitated by greater reliance on ketone bodies, further reduces cellular stress. Their diuretic and natriuretic actions also help to decrease cardiac wall tension and pressure, thereby lessening mechanical stress-induced fibrosis [76,77,78,79].

### 3.4. Impact on Arterial Stiffness, Endothelial Function, and Cardiac Remodeling

Another significant benefit of SGLT2is is their cardioprotective and renoprotective vascular effects. These agents counteract hyperglycemia-induced reductions in nitric oxide synthase expression, thereby improving vasodilation and exerting anti-inflammatory effects on endothelial tissue [72,80,81]. In animal studies, canagliflozin has demonstrated the ability to improve aortic relaxation in response to acetylcholine, while also decreasing oxidative stress and inflammation [82]. Similarly, dapagliflozin improves flow-mediated dilation, decreases aortic stiffness, and lowers the renal resistive index—effects that occur independently of natriuresis or glycemic changes [83].

These vascular benefits have been further supported by proteomic studies. For example, Yue et al. found that empagliflozin resulted in downregulation of enzymes critical to fatty acid metabolism (FASN, SCD3, ACSL1, ACSL5) and enhanced aortic compliance in obese mice. These findings indicate a potential direct effect on vascular lipid regulation and arterial stiffness [84]. Of note, preliminary evidence suggests that SGLT2is may serve as a valuable addition to aortic stenosis management by addressing critical aspects of this valvulopathy, including fibrosis, inflammation, oxidative stress, and metabolic imbalances [85].

## 4. Clinical Evidence Across the CRM Spectrum

SGLT2is have demonstrated their benefits across several conditions that represent components of the CRM syndrome, as evidenced by rigorously conducted clinical trials (Table 1).

### 4.1. Diabetes Mellitus Type 2

The therapeutic benefits of SGLT2is in individuals with T2DM are well established through robust clinical trial data. Among patients with T2DM at high risk for CVD or CKD, SGLT2is have been shown to significantly lower the occurrence of major adverse CV events (MACEs) and reduce the progression of kidney disease [98,99].

The EMPA-REG OUTCOME trial randomized individuals with established CVD and T2DM and was the first large clinical outcome trial of an SGLT2i to report a remarkable CV mortality benefit. Compared to placebo, empagliflozin treatment lowered the risk of the primary composite outcome of MACEs (including CV death, nonfatal MI, and nonfatal stroke) by 14%, reduced CV death risk by 38%, and decreased hospitalization for HF (HHF) by 35%, with consistent effects seen in patients regardless of prior HF history at enrollment [94].

The CV benefits identified in the EMPA-REG OUTCOME trial were later supported by the CANVAS Program, which integrated data from two randomized, placebo-controlled trials—CANVAS and CANVAS-R—and evaluated the effects of canagliflozin in individuals with T2DM at high CVD risk. In the CANVAS Program, canagliflozin significantly reduced the risk of the primary composite outcome of MACEs by 14% relative to placebo. Although canagliflozin did not demonstrate statistically significant reductions in CV mortality or all-cause mortality compared to placebo, it was associated with a 33% decrease in the risk of HHF. These findings further support the CV and HF benefits of SGLT2 inhibition in this high-risk group [96].

The DECLARE–TIMI 58 trial evaluated the CV impact of SGLT2 inhibition in a larger cohort of 17,160 patients with T2DM, most of whom did not have established ASCVD. In this randomized, placebo-controlled trial, dapagliflozin showed no significant effect on reducing MACE risk; however, it significantly decreased the risk of the combined endpoint of CV death or HHF by 17%, largely due to a 27% reduction in HHF. These findings extended the cardioprotective benefits of SGLT2is to a lower-risk population, thereby broadening the scope of their therapeutic indications [95].

### 4.2. Chronic Heart Failure

The aforementioned clinical trials provided initial evidence that SGLT2is significantly reduce hospitalizations due to HF. The DAPA-HF trial was the first large-scale study specifically aimed at assessing the impact of SGLT2is in patients with clearly defined HF. In this trial, individuals with symptomatic heart failure with reduced ejection fraction (HFrEF) were randomized to receive either dapagliflozin or placebo, in addition to standard guideline-directed medical therapy (GDMT). Out of 4744 participants enrolled, 2761 (58%) did not have T2DM at the time of recruitment. Dapagliflozin led to a 26% decrease in the primary composite outcome (CV death, HHF, or urgent HF-related hospitalization requiring intravenous treatment) when compared to placebo. Additionally, patients treated with dapagliflozin had improved HF symptoms, reductions in N-terminal pro-B-type natriuretic peptide (NT-proBNP), and a decrease in overall mortality [86]. This study laid the foundation for SGLT2is to be established as a cornerstone therapy for HFrEF.

The EMPEROR-Reduced trial reinforced the findings of DAPA-HF by including 3730 patients with symptomatic HFrEF, approximately half of whom had T2DM, most receiving standard GDMT. Empagliflozin reduced the primary composite outcome of CV death or HHF by 25% compared to placebo, and also significantly lowered total HHF events by 31%. Although the reductions in CV and all-cause mortality were not statistically significant, the mortality trends aligned with those reported in the DAPA-HF trial. Importantly, the benefits of empagliflozin were consistent across predefined subgroups, regardless of age, sex, or race, including patients with or without T2DM and those with either ischaemic or non-ischaemic forms of HF [87]. The DAPA-HF and EMPEROR-Reduced trials established SGLT2is as part of the GDMT for patients with HFrEF.

HFpEF accounts for approximately 50% of the HF population, and its prevalence increases significantly with age and the presence of cardiometabolic conditions [100]. Before the introduction of SGLT2is, management of HFpEF was largely limited to symptom control and addressing associated comorbidities [101]. The EMPEROR-Preserved trial was designed to evaluate whether the benefits of empagliflozin, previously demonstrated in HFrEF, would also apply to patients with HFpEF (left ventricular ejection fraction [LVEF] > 40%). The results showed a 29% reduction in the risk of hospital readmission due to decompensated HF when compared to placebo, a finding that reached statistical significance. Additionally, empagliflozin led to a significant 21% relative risk (RR) reduction in the combined outcome of CV death or HHF compared to placebo, with the benefit largely driven by a decreased incidence of HHFs in the treatment group [88]. Similarly, the DELIVER trial assessed dapagliflozin in patients with mildly reduced or preserved ejection fraction (LVEF > 40%). Dapagliflozin was found to reduce the primary endpoint—a composite of CV death or worsening HF events—by 18% compared to placebo, and the incidence of HHF was also reduced by 23% [89]. A recent meta-analysis of 18 randomized clinical trials (RCTs) involving patients with HFmrEF or HFpEF demonstrated that treatment with SGLT2is led to significant improvements in health-related quality of life (HRQoL) [14]. Of note, the ongoing SOTA-P-CARDIA trial is examining the potential benefits of sotagliflozin in HFpEF patients, regardless of diabetes status [102].

The effectiveness of SGLT2is in treating HF across the full spectrum of LVEF has been consistently shown in four major RCTs: DAPA-HF, DELIVER, EMPEROR-Reduced, and EMPEROR-Preserved. These results are further reinforced by two meta-analyses—one pooling data from EMPEROR-Reduced and EMPEROR-Preserved (*n* = 9718) to evaluate empagliflozin, and another combining findings from DAPA-HF and DELIVER (*n* = 11,007) to assess dapagliflozin. Both analyses reported significant reductions in the composite outcome of CV death and HHF, with no observed heterogeneity across the full spectrum of LVEF [103,104]. Of note, the beneficial effects of SGLT2is also extend to various forms of HF beyond dilated and ischemic types. A recently published meta-analysis suggests that the use of SGLT2is may improve the prognosis of patients with transthyretin amyloid cardiomyopathy (ATTR-CM) [105].

### 4.3. Acute Heart Failure

The SOLOIST-WHF trial investigated the safety and efficacy of sotagliflozin, a dual SGLT1/2 inhibitor, in patients with T2DM recently hospitalized for worsening HF, focusing on early initiation post-decompensation. The majority of participants (79%) had an ejection fraction < 50%. Although the trial ended early due to funding issues and the COVID-19 pandemic, with a median follow-up of under one year, sotagliflozin was associated with a significant 33% reduction in the primary composite outcome of CV death, total HF hospitalizations, and unplanned HF visits compared to placebo. The beneficial effect was consistent, even among those with HF with preserved ejection fraction (HFpEF) [90].

The EMPULSE trial investigated the impact of empagliflozin in hospitalized patients with acute HF once clinically stable. Empagliflozin-treated patients achieved greater decongestion and body weight reduction, higher levels of hematocrit, and lower NT-proBNP levels than placebo. These findings underscore the potential of empagliflozin as an effective decongestive treatment in patients admitted with acute HF [106].

### 4.4. Chronic Kidney Disease

SGLT2is have demonstrated significant long-term kidney benefits and can alter disease progression in patients with CKD, regardless of their initial glucose levels. The CREDENCE study, which investigated canagliflozin in patients with T2DM and albuminuric CKD, showed a 30% decrease in the combined risk of end-stage renal failure (ESRD), doubling of serum creatinine, or renal/CV death [93]. The DAPA-CKD trial evaluated dapagliflozin in a wider CKD population of 4304 patients, including those without T2DM. Results showed that dapagliflozin reduced the risk of a composite renal outcome—defined as a ≥50% decline in eGFR, onset of ESRD, or death due to renal or CV causes—by 39% compared to placebo. Analysis of secondary outcomes revealed a significant reduction in the combined risk of CV death or HHF, and 31% decrease in all-cause mortality risk compared to placebo. Importantly, the trial was stopped early because of its clear and substantial benefits [91]. The EMPA-KIDNEY trial further confirmed the nephroprotective effects of SGLT2is. Specifically, empagliflozin was shown to significantly lower the risk of the primary composite outcome (progression of kidney disease or CV death) by 28%, regardless of diabetes status. Additionally, patients treated with empagliflozin experienced a slower annual decline in eGFR compared to those receiving placebo, with an average difference of 0.75 mL/min/1.73 m^2^ per year from baseline through the follow-up period [92]. More broadly, a 2021 meta-analysis encompassing 66,601 participants from eight key trials evaluating empagliflozin, canagliflozin, ertugliflozin, dapagliflozin, and sotagliflozin in CKD patients reported a 40% reduction in serious adverse events among those treated with SGLT2is versus placebo, underscoring their effectiveness in slowing CKD progression [107].

### 4.5. Acute Myocardial Infarction

Acute myocardial infarction (MI) remains a leading cause of morbidity and mortality worldwide, and patients continue to have an elevated risk of developing cardiac remodeling and subsequent HF despite significant therapeutic advances [108,109]. Most adjunctive therapies aimed at reducing infarct size have failed to translate into daily clinical practice. Nevertheless, SGLT2is, after showing favorable CV outcomes in HF, represented an appropriate target for additional research in patients with acute MI.

The DAPA-MI and EMPACT-MI trials were designed to evaluate the role of SGLT2is in the development of cardiac remodeling, HF, and clinical outcomes when commenced shortly after acute MI [110,111]. In the DAPA-MI trial, patients without a history of T2DM and HF were randomized to receive dapagliflozin or placebo after their MI presentation. Although significant improvements in cardiometabolic outcomes were observed, this study had no impact on CV death or HHF compared with placebo [110]. Further, the EMPACT-MI trial investigated the effect of empagliflozin versus placebo in patients with acute MI and increased risk of HF. This study also failed to significantly lower the risk of a first HHF or death from any cause [111]. Conversely, the smaller EMI-STEMI study demonstrated that treatment with empagliflozin before percutaneous coronary intervention (PCI) and continued for 40 days in patients with ST-elevation MI (STEMI) was linked to a significantly higher LVEF compared with placebo [112]. Future RCTs are warranted to further elucidate the potential role of SGLT2is in mitigating adverse cardiac remodeling in MI [113].

### 4.6. Hypertension and Vascular Protection

Several studies have evaluated the antihypertensive properties of SGLT2is. The EMPA-REG BP trial showed that empagliflozin effectively lowered blood pressure in individuals with T2DM and hypertension. Both 10 mg and 25 mg doses led to reductions in mean 24 h systolic blood pressure (SBP)/diastolic blood pressure (DBP) compared to placebo, regardless of the number of background antihypertensive medications used. In patients not on any antihypertensives, the 10 mg and 25 mg doses reduced SBP/DBP by −3.89/−2.58 mm Hg and −3.77/−2.45 mm Hg, respectively. Among those receiving one antihypertensive agent, reductions were −4.74/−1.97 mm Hg with 10 mg and −4.27/−1.81 mm Hg with 25 mg. For patients on two or more antihypertensives, decreases were −2.36/−0.68 mm Hg (10 mg) and −4.17/−1.54 mm Hg (25 mg) [97]. The CREDENCE trial, which examined the impact of canagliflozin in patients with T2DM and kidney impairment, showed an approximate 3.5 mmHg decrease in SBP [93]. Further, the SACRA study found that incorporating empagliflozin into the current antihypertensive regimen of older, non-obese patients with T2DM and uncontrolled nocturnal hypertension led to significant reductions in daytime, 24 h, morning home, and clinic SBP (−9.5, −7.7, −7.5, and −8.6 mm Hg, respectively [114]. Moreover, a large meta-analysis reported a modest mean reduction in SBP of 3.46 mmHg with SGLT2is compared to placebo (95% confidence interval [CI]: −3.83, −3.09). However, this analysis found no significant SBP reduction specifically among patients without T2DM treated with these agents [115].

Beyond their impact on blood pressure, SGLT2 inhibitors also provide a clinically important benefit by improving vascular function. This vascular benefit has been supported by a meta-analysis of 11 clinical studies involving 868 participants, which demonstrated a significant enhancement in flow-mediated dilation [116]. Additionally, a review of 24 preclinical studies reported consistent improvements in endothelial dysfunction and provided insight into potential glucose-independent mechanisms underlying the endothelial benefits of SGLT2is [117].

### 4.7. Nonalcoholic Fatty Liver Disease/Nonalcoholic Steatohepatitis

Beyond their effects on the heart and kidneys, SGLT2is have demonstrated potential benefits in nonalcoholic fatty liver disease (NAFLD) and nonalcoholic steatohepatitis (NASH). NAFLD/NASH are characterized by excessive fat in the liver. Obesity and T2DM, two basic components of CRM syndrome, are strongly linked to these conditions and can contribute to the progression of severe hepatic fibrosis [118,119]. Studies utilizing magnetic resonance imaging (MRI) to assess liver fat have reported reductions in steatosis ranging from 13% to 25% following treatment with SGLT2is, with all studies reaching statistical significance [120,121,122].

### 4.8. Effects of SGLT2 Inhibitors in Patients Without Diabetes Mellitus

Emerging data have increasingly demonstrated that SGLT2is exert favorable CV and metabolic effects even in patients without T2DM. This is further supported by a meta-analysis of eight RCTs that included 5233 patients without T2DM. In this analysis, patients with HF who received an SGLT2i experienced a 20% RR reduction in CV mortality and HHF compared to those who did not receive the agent. In addition, SGLT2i-treated patients had significant reductions in body weight (−1.21 kg), body mass index (BMI) [−0.47 kg/m2], SBP (−1.90 mm Hg), and fasting plasma glucose levels (−0.38 mmol/L) [123].

Regarding CKD, available data strongly supports the use of SGLT2is in patients with CKD who exhibit hyperfiltration-related conditions, such as T2DM, obesity, or HF. However, their efficacy in CKD patients with other disease mechanisms, such as non-obese individuals without T2DM, remains uncertain, as this population was not adequately represented in the large RCTs that established the renoprotective properties of SGLT2is [124]. Further studies are needed to definitively establish the beneficial effects of these agents on kidney function in underrepresented populations.

## 5. Real-World Data and Translational Insights

### 5.1. Observational Studies

Key findings from the landmark RCTs EMPA-REG OUTCOME, CANVAS, and DECLARE-TIMI 58, and the kidney outcome trials CREDENCE, EMPA-KIDNEY, and DAPA-CKD, have clearly established the cardio-renal protective properties of SGLT2is in populations with standardized characteristics. In contrast to RCTs, which follow predefined criteria, observational studies offer cost-effective and inclusive insights from a broader range of populations and clinical settings [125].

Several real-world evidence studies across diverse populations have consistently shown that SGLT2is significantly reduce the risk of HHF, all-cause death, and MACEs in patients with T2DM. In a retrospective observational cohort study conducted in the United States (U.S.) among patients receiving routine care over a 30-month period, canagliflozin was associated with a 30–49% lower risk of HHF compared with dipeptidyl peptidase-4 (DPP-4) inhibitors (hazard ratio [HR]: 0.70, 95% CI: 0.54–0.92), GLP-1 receptor agonists (HR: 0.61, 95% CI: 0.47–0.78), and sulfonylureas (HR: 0.51, 95% CI: 0.38–0.67) [126]. The EASEL study, which examined patients with established CVD and T2DM, found that SGLT2is reduced the risk of MACE (HR: 0.57, 95% CI: 0.50–0.65), all-cause death (HR: 0.57, 95% CI: 0.49–0.66), and HHF (HR: 0.57, 95% CI: 0.45–0.73) by 33–43%, but were associated with a higher risk of below-knee lower extremity amputation [127]. In the OBSERVE-4D study, a meta-analysis of four observational databases including over 700,000 individuals with T2DM, canagliflozin was associated with a 61% lower risk of HHF compared to newly initiated non-SGLT2i therapies in the general population (HR: 0.39; 95% CI: 0.26–0.60). Among patients with established CVD, the risk was reduced by 56% (HR: 0.44; 95% CI: 0.36–0.54), with no observed increase in amputation risk [128]. Additionally, the CNODES study in Canada found 24–57% risk reductions in MACE, CV death, MI, HHF, and all-cause death among over 200,000 new SGLT2i users versus DPP-4 inhibitor users [129]. In a Scandinavian cohort study of 41,966 patients with T2DM (mostly on dapagliflozin), SGLT2is reduced the risk of HHF and all-cause death by 34% (HR: 0.66, 95% CI: 0.53–0.81) and 20% (HR: 0.80, 95% CI: 0.69–0.92), respectively, compared with DPP-4 inhibitor treatment [130]. Similarly, serious renal events were reduced by 58% (HR 0.42, 95% CI 0.34–0.53) with the use of SGLT2is compared with DPP-4 inhibitors, with lower rates of renal replacement therapy and renal-related hospital admissions, while mortality due to renal causes remained comparable between groups [131].

The multinational, observational CVD-REAL (Comparative Effectiveness of Cardiovascular Outcomes in New Users of SGLT2 Inhibitors) study found, after propensity score matching, that SGLT2i use was linked to a reduced risk of death in patients with and without CVD (HR: 0.56; 95% CI: 0.44–0.70 and HR: 0.56; 95% CI: 0.50–0.63, respectively). Additionally, the risk of HF was lower in both groups (HR: 0.72; 95% CI: 0.63–0.82 in those with CVD and HR: 0.61; 95% CI: 0.48–0.78 in those without CVD). The combination of HF or death was also significantly reduced regardless of CVD status (HR: 0.63; 95% CI: 0.57–0.70 and HR: 0.56; 95% CI: 0.50–0.62, respectively) [132]. The subsequent CVD-REAL 2 study, which included patients with T2DM from regions such as the Asia Pacific, the Middle East, and North America, examined a wider spectrum of CV outcomes and patient characteristics. Starting treatment with an SGLT2i was linked to significant risk reductions across multiple CV outcomes: a 49% decrease in the risk of ACD (HR: 0.51, 95% CI: 0.37–0.70), a 36% reduction in HHF (HR: 0.64, 95% CI: 0.50–0.82), a 19% lower risk of MI (HR 0.81; 95% CI: 0.74–0.88), and a 32% reduction in stroke risk (HR: 0.68, 95% CI: 0.55–0.84) [133]. The CVD-REAL 3 study examined renal outcomes in new users of SGLT2is compared to users of other glucose-lowering therapies across five countries: Israel, Italy, Japan, Taiwan, and the United Kingdom. SGLT2i use was associated with a slower decline in eGFR, showing a yearly slope difference of 1.53 mL/min/1.73 m^2^ (95% CI: 1.34–1.72). Over an average follow-up of 14.9 months, patients on SGLT2is had a 51% reduced risk of experiencing the composite kidney outcome compared to those on other glucose-lowering drugs (HR: 0.49; 95% CI: 0.35–0.67), with consistent findings across all participating countries [134].

Lastly, the EMPRISE studies across the US, East Asia, and Europe showed that empagliflozin significantly reduces the risk of HHF, ACD, ESRD, and MACEs compared to DPP-4 inhibitors and GLP-1 receptor agonists. These benefits were observed consistently in patients regardless of baseline CV or kidney disease. Importantly, empagliflozin also slowed the progression of kidney function decline and lowered stroke risk, with more pronounced effects seen in older individuals and those with a history of CVD or HF [135,136,137].

Based on the aforementioned evidence from large RCTs and observational studies which enrolled patients from all over the world, there is now a broad consensus in the medical community that SGLT2is represent a paradigm shift in the management of CRM conditions affecting millions of people.

### 5.2. Cardiac Imaging Studies

HF progression involves changes in the heart’s shape, function, and structure, a process known as cardiac remodeling [138]. Cardiac magnetic resonance (CMR) is the gold standard for visualizing these changes, including volumes, mass, and function, and it uniquely provides tissue characterization of the myocardium [139]. Emerging data underscore the utility of CMR imaging in elucidating the potential cardiac effects of SGLT2is on cardiac remodeling [140]. CMR studies have shown that SGLT2is reduce LV mass in patients with T2DM, suggesting reverse cardiac remodeling in this population, although it remains unclear whether this reflects changes in cardiomyocytes, the interstitium, or their combination [141,142]. In a broader population, a recent meta-analysis including 1008 patients from 23 studies found that SGLT2is treatment significantly reduced LV end-diastolic volume (−7.10 mL; 95% CI: −13.01 to −1.19, *p* = 0.023), LV mass (−4.24 g; 95% CI: −7.88 to −0.60, *p* = 0.027), and epicardial adipose tissue (EAT) (−4.94 mL; 95% CI: −9.06 to −0.82, *p* = 0.019). LV stroke volume was improved in a subgroup analysis in patients with reduced LVEF [140].

Advancements in CMR imaging technology now allow for the assessment of extracellular volume (ECV), which is considered a reliable marker of LV fibrosis [143]. Manson et al. showed that six months of empagliflozin treatment in patients with T2DM and coronary artery disease (CAD) led to reductions in both extracellular volume (ECV) and indexed LV mass, while indexed intracellular volume and fibrosis biomarkers—such as soluble ST2 (sST2) and matrix metalloproteinase-2 (MMP-2)—remained unchanged [144]. Furthermore, a multicenter, double-blind, placebo-controlled trial enrolled 100 patients with HFpEF and T2DM who were randomly assigned to receive either dapagliflozin or placebo for 12 months. Dapagliflozin treatment significantly reduced myocardial fibrosis, as measured by CMR-derived ECV, and led to improvements in LV mass index, glycemic control, and exercise tolerance [145]. Conversely, the previously discussed meta-analysis did not show any impact of SGLT2i treatment on tissue characterization indices, such as T1 mapping and ECV, across the entire population. These findings should be approached cautiously, given the limited number of studies assessing these parameters and the possibility of publication bias affecting ECV results. Until proven otherwise, the reduction in LV mass observed with SGLT2i treatment appears to result mainly from LV unloading rather than a reduction in ECV [140]. To reach a final conclusion, additional studies are currently underway to evaluate the effects of SGLT2is on ECV (NCT03782259, NCT04490681).

Looking ahead, CMR imaging holds considerable promise for advancing personalized medicine in CRM syndrome. It can aid in early disease diagnosis, in deciding when to start SGLT2i, and in monitoring response, ultimately improving patient outcomes. Continuous research efforts are required to refine when CMR is most helpful in CRM syndrome, identify which individuals are the best candidates, and determine how it can assist physicians in deciding when to start SGLT2i.

### 5.3. Biomarker-Based Evidence

Numerous biomarkers of CV and renal function are affected by SGLT2is. Patients with CKD often exhibit increased levels of cardiac troponin (cTn), which serve as indicators for both CV and overall mortality risks within this group [146]. Several studies have shown that SGLT2is can hinder the progression or reduce levels of cTn in individuals with or without T2DM across various CV settings [147,148,149]. However, Griffin et al. indicated no significant effect in certain populations, such as patients with T2DM and stable HF [150].

Natriuretic peptides, including B-type natriuretic peptide (BNP) and N-terminal pro B-type natriuretic peptide (NT-proBNP), are commonly used biomarkers for the diagnosis and management of acute and chronic HF, and have also been shown to predict CV risk in patients with CKD [151]. Moreover, plasma levels of natriuretic peptides tend to rise as kidney function deteriorates and are linked to an increased risk of advancing to ESRD [152]. SGLT2is have consistently shown to reduce NT-proBNP levels in patients with HF, particularly those with HFrEF. These effects were observed in both diabetic and non-diabetic populations, including patients with or without recent MI [153,154,155,156,157,158]. However, results vary across studies, with some showing no significant effect in patients with stable HF or those with HFpEF [150,159,160,161]. Variations in outcomes may be due to differences in comorbidities, DM duration, and age [162].

The exploration of new molecular biomarkers remains highly relevant, as they offer the potential for early detection of target organ damage. Galectin-3 is an inflammatory mediator belonging to the β-galactoside-binding lectins, secreted by activated macrophages. Galectin-3 promotes collagen deposition, particularly in the heart and kidney, and causes fibrosis when bound to the extracellular matrix. Indeed, elevated galectin-3 levels are associated with an accelerated decline of GFR [163,164]. Patients with T2DM and higher galectin-3 levels appeared to derive greater benefit from dapagliflozin treatment in reducing kidney disease progression [165].

SST2 is a member of the IL-1 receptor family, and it is released by vascular endothelial cells, cardiomyocytes, and cardiac fibroblasts when exposed to stress and/or damage [166,167]. Elevated sST2 levels have been associated with increased risk of HHF, morbidity, and mortality [168,169]. In patients with T2DM and CVD, canagliflozin slowed the rise in sST2 levels over a six-year period. Notably, patients with baseline sST2 levels above 35 ng/mL experienced greater reductions in MACEs [147]. However, this effect has not been confirmed in older T2DM patients compared to placebo [148].

Tubular injury biomarkers, including kidney injury molecule-1 (KIM-1), neutrophil gelatinase-associated lipocalin (NGAL), and liver-type fatty acid-binding protein (L-FABP), are proteins secreted into the bloodstream by tubular epithelial cells following damage. SGLT2i treatment has demonstrated heterogeneous effects on these markers [162]. Dapagliflozin was associated with a reduction in KIM-1 levels and no effect on L-FABP or NGAL levels [170]. Of note, elevated serum and urinary NGAL levels in patients on SGLT2is during acute illness and acute kidney injury (AKI) suggest possible distal tubular injury linked to medullary hypoxia. In contrast, unchanged KIM-1 levels may indicate preserved cortical oxygenation [171].

As new biomarkers related to CRM syndrome are discovered and the use of SGLT2is is expected to significantly increase in this clinical setting, the need to explore the interactions between these agents and biomarkers becomes crucial. Understanding these interactions is key to optimizing treatment strategies and improving patient outcomes. Toward this goal, further human trials are needed to clarify the scope and nature of the connection between SGLT2i use and changes in CRM-related biomarkers.

## 6. Positioning SGLT2 Inhibitors in Contemporary Therapeutic Algorithms

### 6.1. Integration with RAAS Inhibitors, GLP-1 Receptor Agonists, and MRAs

SGLT2is are frequently combined with other therapeutic agents—such as RAAS inhibitors, mineralocorticoid receptor antagonists (MRAs), and glucagon-like peptide-1 receptor agonists (GLP-1 RAs)—to enhance CV and renal outcomes, leveraging shared pathophysiological pathways that may provide broad benefits across the stages of CKM syndrome.

Angiotensin-converting enzyme inhibitors (ACEIs) and angiotensin receptor blockers (ARBs) are the most frequently prescribed RAAS inhibitors in clinical practice. These medications have demonstrated effectiveness in lowering the incidence of ESKD and MACEs in patients with diabetic kidney disease (DKD) [172]. ACEIs or ARBs combined with SGLT2is may exert synergistic effects through various mechanisms, including improved insulin sensitivity, reduced systemic oxidative stress, and attenuation of inflammatory responses. Additionally, ACEIs/ARBs may counteract the afferent arteriole vasoconstriction induced by SGLT2is, thereby improving clinical outcomes in DKD [173]. A large meta-analysis of RCTs demonstrated that combination therapy with an ACEI or ARB and an SGLT2i significantly reduced the risk of MACE (HR: 0.88, 95% CI: 0.82–0.94; I^2^ = 44.9%), CV death or HHF (HR: 0.75, 95% CI: 0.70–0.81; I^2^ = 34.6%), and composite kidney outcomes (HR: 0.59, 95% CI: 0.54–0.66; I^2^ = 0%) in comparison with ACEI or ARB alone in individuals with T2DM [174].

Several CV outcomes trials (CVOTs) have consistently shown that GLP-1 RAs reduce atherosclerotic events, including non-fatal MI, non-fatal stroke, and CV death [175,176]. These agents have a positive impact on several CV risk factors, such as obesity, hypertension, and dyslipidemia [176]. Emerging evidence underscores the promising role of GLP-1 RAs in treating HFpEF, particularly in patients where obesity is a contributing factor. A recent meta-analysis of six randomized controlled trials, including a total of 8788 patients with HFmrEF or HFpEF, demonstrated that treatment with GLP-1 RAs led to a significant reduction in the composite outcome of CV death or worsening HF events [177]. Regarding their renoprotective effects, the FLOW trial involving 3533 patients with T2DM and CKD demonstrated that semaglutide reduced the incidence of the primary outcome by 24% compared to placebo, with consistent positive effects seen in kidney-related outcomes, CV death, eGFR decline, MACE, and overall mortality [178].

Furthermore, tirzepatide, a novel dual GIP/GLP-1 RA, has shown remarkable efficacy in the management of obesity, in delaying the onset of T2DM among individuals with prediabetes, in reducing HbA1c levels, and improving obesity-related obstructive sleep apnea (OSA) [179,180].

SGLT2is and GLP-1 RAs may synergistically improve CV and renal outcomes by targeting complementary mechanisms. Both classes help decrease oxidative stress, inflammation, and neurohormonal activation, while improving endothelial function and metabolic efficiency [78,79,154,181,182,183]. To evaluate the potential synergistic effects of GLP-1 RAs and SGLT2is, Patel et al. carried out a retrospective cohort study including adult patients with T2DM, body mass index (BMI) ≥ 27 kg/m^2^, and HFpEF who were receiving SGLT2i therapy. After propensity score matching, 7044 patients remained in each cohort. The cohort which received the combination therapy with GLP-1 RA and SGLT2i demonstrated a significantly lower risk of HF exacerbations (−9%), overall unplanned emergency department attendance or admissions to hospital (−8%), new-onset atrial arrhythmias (−1%), new-onset AKI (−6%), and pulmonary hypertension (−2%), compared to the SGLT2i-only cohort. Additionally, the investigators found modest reductions in C-reactive protein (CRP) levels (−1%) and need for renal replacement therapy (−1%). These clinical benefits were consistent across various BMI categories and ejection fraction values, and were also observed in patients with elevated natriuretic peptide levels [184]. A sub-analysis of the FLOW trial explored the renoprotective effects of semaglutide in individuals with T2DM and CKD, categorized according to baseline SGLT2i use. Among participants already on SGLT2is, the primary composite outcome—comprising kidney failure, a ≥50% reduction in eGFR, kidney-related death, or CV death—was observed in 14.8% (41/277) of those treated with semaglutide compared to 13.9% (38/273) in the placebo group (HR 1.07; 95% CI: 0.69–1.67; *p* = 0.755). In contrast, among those not receiving SGLT2is, the outcome occurred in 19.5% (290/1490) of the semaglutide group versus 24.9% (372/1493) of those on placebo (HR 0.73; 95% CI: 0.63–0.85; *p* < 0.001). This study showed that benefits of semaglutide were observed in both participants who were taking SGLT2is at baseline and those who were not, indicating potential independent renal benefits [185].

A recent population-based cohort study found that combination therapy with GLP-1 RA and SGLT2i was linked to a 30% reduction in the risk of MACEs compared to GLP-1 RA alone (7.0 vs. 10.3 events per 1000 person-years; HR: 0.70, 95% CI: 0.49–0.99), and a 57% decrease in serious renal events (2.0 vs. 4.6 events per 1000 person-years; HR: 0.43, 95% CI: 0.23–0.80). Compared with SGLT2i alone, the combination was associated with a 29% lower risk of MACEs (7.6 vs. 10.7 events per 1000 person-years; HR: 0.71, 95% CI: 0.52–0.98), whereas the reduction in serious renal events was associated with a wide CI (1.4 vs. 2.0 events per 1000 person-years; HR: 0.67, 95% CI: 0.32–1.41) [186].

Patients with HF are frequently treated with SGLT2is and MRAs. Given their different mechanisms of action, the combination of these agents may improve clinical outcomes through additive effects.

In the FINEARTS-HF trial, 6001 patients with HF and a LVEF ≥ 40% were studied to evaluate the effects of finerenone. Of these, 817 (13.6%) were receiving SGLT2i. Over a median follow-up period of 2.6 years, finerenone treatment reduced the risk of the primary outcome to a similar extent in participants both receiving SGLT2i therapy (RR: 0.83, 95% CI: 0.60–1.16) and those not on SGLT2i at baseline (RR 0.85, 95% CI: 0.74–0.98; P_interaction_ = 0.76). Although fewer patients initiated SGLT2i during follow-up in the finerenone group, time-updated analyses showed that SGLT2i use did not alter the benefit of finerenone. These findings suggest that combined use of finerenone and SGLT2is may offer additive CV protection in patients with HFmrEF and HFpEF [187].

In CKD, according to a recent meta-analysis addressing the effect of SGLT2is/MRAs combination on albuminuria, blood pressure, eGFR, and serum potassium, which included four randomized trials and 272 patients, it was found that patients receiving the combination had significantly greater reductions in albuminuria (−33.6%) and SBP (−6.1 mm) compared to SGLT2i alone [188]. A combined analysis of the CREDENCE (*n* = 4401) and FIDELIO-DKD (*n* = 5734) trials demonstrates the significant added benefit of finerenone when used alongside SGLT2is. This dual therapy was linked to a 50% lower risk of a composite outcome, which included doubling of serum creatinine, progression to ESKD, or kidney failure-related death (HR: 0.50; 95% CI: 0.44–0.57). For a 50-year-old patient, this corresponded to an event-free survival of 16.7 years (95% CI: 18.1–21.0) with the combination treatment, compared to 10.0 years (95% CI: 6.8–12.3) with standard care involving ACEIs or ARBs, reflecting an additional 6.7 years (95% CI: 5.5–7.9) of event-free survival [189].

A recent study highlights the substantial benefits of triple therapy combining SGLT2is, finerenone, and GLP1 RAs in patients with T2DM and a urinary albumin-to-creatine ratio (UACR) ≥ 30 mg/g. In comparison to RAAS inhibitor monotherapy, this combination significantly lowered the risk of MACE, including non-fatal MI, stroke, and CV death (HR: 0.65, 95% CI: 0.55–0.76). For a 50-year-old patient initiating triple therapy, the estimated MACE-free survival extended by 3.2 years (21.1 vs. 17.9 years), along with further improvements such as prolonged survival without HHF (3.2 years), delayed CKD progression (5.5 years), reduced CV death (2.2 years), and decreased all-cause mortality (2.4 years). These findings emphasize the potential of comprehensive, multi-drug approaches in managing patients with T2DM and early signs of kidney damage [190].

### 6.2. Guidelines from ADA, ESC, KDIGO, and ACC

SGLT2is are highly recommended across most major guidelines for CV, renal, and diabetic conditions due to their proven efficacy in large RCTs. The 2024 KDIGO (Kidney Disease: Improving Global Outcomes) guidelines strongly recommend initiating SGLT2is in patients with T2DM and CKD who have an eGFR ≥ 20 mL/min/1.73 m^2^ (IA). Once initiated, it is considered reasonable to continue SGLT2i therapy even if eGFR falls below 20 mL/min/1.73 m^2^, unless poorly tolerated or kidney replacement treatment is started. These updated guidelines also expand the recommendation to include individuals with CKD from causes other than DM. Specifically, KDIGO now strongly recommends treatment with an SGLT2i in non-diabetic adults with CKD who have an eGFR ≥ 20 mL/min/1.73 m^2^ and a UACR ≥ 200 mg/g (≥20 mg/mmol), or in those with HF, regardless of albuminuria level (IA). For adults with an eGFR of 20–45 mL/min/1.73 m^2^ and a UACR < 200 mg/g (<20 mg/mmol), SGLT2is are suggested as a treatment option (2B). This recommendation reflects a strong consideration of the potential long-term benefits of SGLT2i therapy in individuals without T2DM but with significantly reduced kidney function, aiming to lower the risk of kidney failure. However, it also acknowledges the remaining uncertainty in this population due to the relatively short duration of follow-up in available RCTs [191].

The American Diabetes Association (ADA) Standards of Care in Diabetes emphasize the use of cardioprotective and renoprotective therapies in adults with T2DM, independent of glucose control considerations. In individuals with established or high risk of ASCVD, ADA suggests that the treatment plan should include agents with proven CV benefit—specifically, GLP-1 RAs and/or SGLT2is—to reduce MACEs (A). For those with HF (regardless of EF), SGLT2is are recommended for both glycemic control and reduction in HFF (A). In CKD, it is strongly recommended that either an SGLT2i or a GLP-1 RA with established efficacy in this population be incorporated to manage hyperglycemia, slow CKD progression, and reduce CV risk (A). Notably, while the glycemic efficacy of SGLT2is declines at eGFR levels below 45 mL/min/1.73 m^2^, their CV and renal benefits remain clinically relevant [192].

The European Society of Cardiology (ESC) Guidelines for managing CVD in patients with T2DM offer evidence-based recommendations for preventing and treating CV complications, reflecting data available through January 2023. These guidelines strongly endorse the use of SGLT2is in all patients with HFrEF and T2DM to lower the risk of HHF and CV death (IA). Additionally, empagliflozin and dapagliflozin are advised for patients with T2DM and an LVEF greater than 40% to reduce the risk of HHF or CV death (IA). Consistent with KDIGO recommendations, initiating SGLT2is is strongly recommended in patients with T2DM and CKD with an eGFR ≥ 20 mL/min/1.73 m^2^ to decrease the risk of CVD and kidney failure (IA). SGLT2is are also recommended in patients with T2DM and multiple ASCVD risk factors or established ASCVD to reduce the risk of HFF (IA). Of note, these guidelines suggest switching glucose-lowering therapy from agents without proven CV benefit to those with proven CV benefit, such as SGLT2is [193].

The 2021 ESC Guidelines for managing acute and chronic HF strongly endorse dapagliflozin or empagliflozin for all patients with HFrEF to lower the risk of HHF and mortality (IA) [194]. In a 2023 focused update that incorporated results from the EMPEROR-Preserved and DELIVER trials, this recommendation was expanded to include patients with HFmrEF and HFpEF (IA) [195]. Additionally, the 2024 ESC Guidelines on chronic coronary syndromes highlight the cardioprotective benefits of SGLT2is in reducing adverse events in ASCVD. They recommend SGLT2i therapy with proven CV benefits for patients with T2DM and chronic coronary syndrome to decrease CV events, regardless of baseline or target HbA1c levels and irrespective of other glucose-lowering treatments (IA) [196].

According to 2023 American College of Cardiology (ACC) guidelines for the management of patients with chronic coronary disease (CCD), in patients with CCD and T2DM, the use of an SGLT2i with proven CV benefit is recommended to reduce the risk of MACEs. In this population, adding an SGLT2i is projected to provide intermediate value compared to standard care, based on U.S. cost-effectiveness analyses. Additionally, for patients with CCD and HFrEF, SGLT2is are strongly recommended regardless of DM status, as they reduce CV death and HFF while improving quality of life (IA). These benefits make their addition to GDMT of intermediate value at U.S. prices. In those with HF and LVEF > 40%, SGLT2i can also be beneficial by decreasing HHF and improving quality of life (2A); however, their overall value in this group at current U.S. prices is considered uncertain. Moreover, in patients with T2DM and established CVD or high CV risk, SGLT2is are also recommended to prevent HHF [197].

In patients with HF, the 2022 ACC guidelines recommend SGLT2is for those with symptomatic chronic HFrEF or HF with coexisting T2DM, to reduce CV mortality, HF-related morbidity, and to aid in glycemic control, regardless of diabetes status (IA). In patients with T2DM and either established CVD or at high CV risk, SGLT2i should also be used to prevent HHF (IA) [198].

While the current aforementioned guidelines are helpful in daily clinical practice, they primarily focus on risk stratification and management of individual conditions such as T2DM, kidney disease, or CVD, rather than adopting a comprehensive approach to CRM syndrome management. This underscores the need for integrated guidelines that address the syndromic and interconnected nature of CRM syndrome pathophysiology.

### 6.3. Safety of SGLT2 Inhibitors

Despite the significant benefits of the SGLT2is in the context of CRM syndrome, clinicians must carefully consider their potential side effects to ensure that therapeutic benefit consistently outweighs the risks. The predominant adverse event associated with SGLT2i therapy is genitourinary tract infections [199,200]. SGLT2i users appear to have a three- to sixfold higher risk of developing this complication compared with non-users, primarily as a consequence of enhanced glucosuria [53,199,200].

A less common but clinically significant adverse event is euglycemic diabetic ketoacidosis (euDKA), which results from increased ketone production combined with enhanced renal reabsorption. It is often precipitated by triggers such as infection, sepsis, trauma, or fasting [201,202]. Of note, a recent meta-analysis has not demonstrated an overall increase in euDKA risk with SGLT2i use [203]. Concerns regarding a possible association with amputations and fractures, initially raised in the CANVAS program, have also not been confirmed in subsequent meta-analyses [96,204]. Moreover, SGLT2is have been linked to an increased risk of volume depletion, although this does not appear to translate into a higher incidence of AKI [200]. Finally, although cases of Fournier’s gangrene have been reported, large case–control analyses and data from the DECLARE-TIMI trial do not support a statistically significant association [205].

Given that the use of SGLT2is is expected to increase over time, strategies to prevent or mitigate their adverse effects should be implemented. Such strategies should include proper genital hygiene, routine foot examination, reduction in diuretic use to prevent volume depletion, and patient education on recognition of the early signs of euDKA.

### 6.4. Sex-Based Differences in SGLT2i Use

Emerging data indicate that there are significant sex-based differences in the effects of SGLT2is on the components of the CRM syndrome. Indeed, a recently published meta-analysis showed that these agents lower the risk of primary composite outcomes (CV death and HHF) in patients with HF, irrespective of sex, though the effect was weaker in women [206]. Similarly, another meta-analysis reported that treatment with SGLT2is in women with T2DM was associated with a smaller reduction in MACE compared to men [207]. These differences may be attributed to multiple factors—the clinical profile of women with HF and DM, the female proportion in major HF and DM trials, unique aspects of female physiology, the biological effect of sex hormones, socio-cultural differences, and the pharmacodynamics of SGLT2is [206,207]. Regarding CKD, a recent study revealed a significant gender disparity, with women with CKD receiving SGLT2is less frequently than men. Of note, women are less likely to be diagnosed early with CKD, seek medical attention, and receive guideline-recommended medication [208].

### 6.5. Multidisciplinary Implications for Primary Care, Cardiology, Nephrology, Endocrinology, and Geriatrics

SGLT2is are a breakthrough therapy with broad clinical utility in the management of T2DM, HF, and CKD, with growing interest in their role in treating non-alcoholic fatty liver disease (NAFLD) and promoting weight loss [52,209]. Due to their multifaceted actions, these agents have been proposed as potential therapeutic agents in the management of CRM syndrome. However, despite robust evidence of their remarkable cardio-renal-metabolic benefits, SGLT2is remain underutilized, especially among patients without T2DM [16]. Barriers identified include lack of familiarity with the emerging clinical entity of CRM syndrome and with the indications for SGLT2is in patients without T2DM, and in older or frail patients. There is also concern regarding the occurrence of side effects such as genitourinary infections and diabetic ketoacidosis. To address these issues, targeted physician education on the expanded indications and safety profile of SGLT2is is essential, along with fostering collaboration among primary care physicians, cardiologists, nephrologists, endocrinologists, and geriatricians to optimize the management of CRM syndrome. Early and comprehensive integration of SGLT2is across all relevant indications enables healthcare providers to actively slow the advancement of CRM syndrome and mitigate complications in patients with existing diseases.

## 7. Future Perspectives

CRM syndrome has garnered significant attention in recent years, as it represents a serious public health concern associated with substantial adverse outcomes. A growing body of evidence has enhanced our understanding of the complex interplay between the heart, kidneys, and metabolic disorders, revealing multiple pathophysiological mechanisms involving diverse cellular pathways. Nevertheless, the pathophysiology of CRM syndrome remains only partially understood and continues to be the subject of ongoing investigation.

At present, we are reasonably effective in preventing and managing each component of the CRM syndrome individually; however, several critical knowledge gaps remain regarding the optimal strategies for addressing this clinical entity as a whole. Key areas of uncertainty include optimal models for interdisciplinary CRM care, strategies for early-life prevention, long-term approaches to obesity pharmacotherapy, and the management of CV disease in advanced CKD [19].

SGLT2is, due to their multifaceted therapeutic benefits in CV, renal, and metabolic diseases, have emerged as a pillar for CRM syndrome. Landmark RCTs have robustly established these effects across a wide spectrum of patients, regardless of diabetes status, an important factor given the significant heterogeneity among individuals in CRM syndrome. However, large, well-designed RCTs are required to definitively establish the potential effects of SGLT2is at each specific stage of the syndrome. Moreover, mechanistic investigations are needed to elucidate the primary pathways through which SGLT2is exert their off-target effects, particularly in non-diabetic and prediabetic populations.

There is compelling evidence that the combination of SGLT2is and GLP-1 RAs represents a promising strategy to modify the development and progression of CRM syndrome. This arises from the fact that each agent acts on distinct facets of the syndrome. SGLT2is demonstrate more pronounced benefits in providing renal protection and in managing HF, whereas GLP-1 RAs are more effective in promoting weight loss, reducing glucose levels, and offering CV protection. However, at present, there are no clear guidelines regarding the timing of combination therapy or the specific clinical indications for its use. Further research is required to address these issues.

An additional field that warrants further investigation is the use of SGLT2is in patients with advanced CKD. According to current guidelines, initiation of these agents is recommended in appropriately selected patients only when eGFR ≥ 20 mL/min/1.73 m^2^ [191]. Nevertheless, a recently published observational study provided preliminary evidence that SGLT2is may also be beneficial in patients with T2DM and stage 5 CKD (eGFR ≤ 15 mL/min/1.73 m^2^), without increasing the risks of early dialysis initiation or infection [210]. To establish definitive conclusions, however, large RCTs are required.

Furthermore, the benefits of SGLT2is in older and frail adults with T2DM and HF remain mostly uncertain, primarily due to the underrepresentation in clinical trials [211]. It is encouraging, however, that more studies are underway to address this issue. A recent meta-analysis suggested that these agents reduced total mortality, HHF events, and cardiac death in older or frail patients with T2DM and HF, though they did not appear to affect macrovascular or renal outcomes [212]. Notably, existing data suggest that these agents are well tolerated in this population [211,213]. As the proportion of older adults continues to rise due to population ageing, there is a compelling need for additional evidence on CRM management in this population.

Given the coexistence of multiple conditions within this syndrome, along with their complex interactions and the numerous variables that affect patient’s outcomes, artificial intelligence (AI) has the potential to serve as a valuable tool for clinicians. Machine learning algorithms applied to CRM syndrome can support precise risk stratification and early detection of disease progression, thereby improving prognosis.

Several trials are currently underway to provide new insights into the treatment of CRM syndrome with SGLT2is, including the CONFIDENCE trial (NCT05254002), which is designed to recruit a broad group of patients with CKD and T2DM, and will examine whether starting finerenone together with a SGLT2i provides greater renal protection compared with initiating either agent alone [214]; the PRECIDENTD trial (NCT05390892), which will assess SGLT2is and GLP-1 RAs by comparing their effects on the cumulative occurrence of CV events, kidney-related outcomes, and mortality in patients with T2DM who have established ASCVD or are at high risk of developing it [215]; and the RENAL LIFECYCLE trial, which will examine the efficacy and safety of dapagliflozin in patients with severe CKD (NCT05374291). Finally, the EMPA-AF trial is designed to evaluate the effects of empagliflozin in patients with atrial fibrillation who also have DM or are over-weight and have HF (NCT04583813).

## 8. Integrative Treatment Strategies for Cardio-Renal-Metabolic Syndrome: The Role of SGLT2 Inhibitors

Taken together, robust evidence supports SGLT2is as cornerstone therapy for CRM syndrome. These agents, either as monotherapy or in combination with GLP-1 RAs or GIP/GLP-1 RAs, have the potential to address most components of the syndrome. It is important to acknowledge, however, that much of the evidence regarding the use of SGLT2is derives from recent studies and may not yet be reflected in current guidelines. Nevertheless, given the pandemic scale of the problem, timely action is imperative.

Based on the staging of the CRM syndrome, SGLT2is have no established role in Stage 0, where management primarily focuses on education and the promotion of a healthy lifestyle to maintain CV health. In Stage 1 our target is the reduction in excess adiposity through lifestyle modifications, with anti-obesity medications considered when comorbidities are present and weight loss goals are not achieved. Although SGLT2is have been shown to promote weight loss, they are not recommended at this stage to be given for this purpose alone, but rather as a supplementary therapy alongside GLP-1 RAs or GIP/GLP-1 RAs.

**Figure 2:**
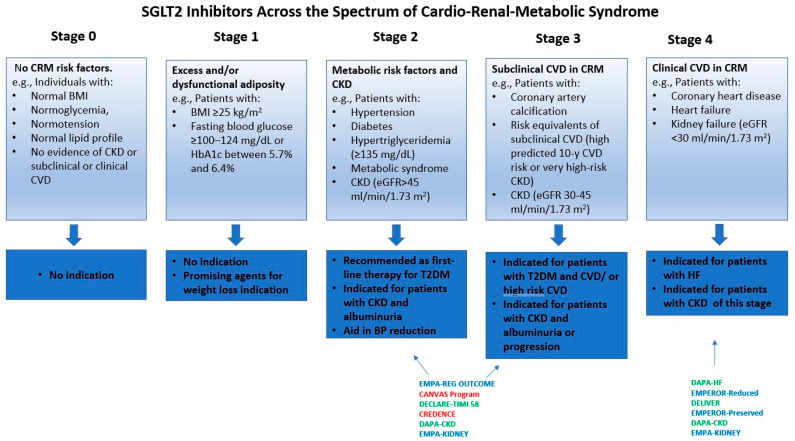
Within the CRM framework, SGLT2is have no indication in Stages 0 and 1 but become highly relevant from Stage 2 onward. In patients with T2DM or CKD (Stage 2), SGLT2is consistently reduce HF events and slow kidney disease progression, with benefits observed even in patients without T2DM. In Stage 3, early initiation is warranted in patients with T2DM given the high absolute risk. In Stage 4, these agents reduce HF events across the EF spectrum and slow CKD progression, contributing to fewer composite cardiovascular–renal outcomes. Trials in red studied canagliflozin; blue, empagliflozin; green, dapagliflozin. SGLT2, sodium-glucose cotransporter 2; CRM, cardio-renal-metabolic; CKD, chronic kidney disease; CVD, cardiovascular disease; BMI, body mass index; HbA1c, hemoglobin A1C; eGFR, estimated glomerular filtration rate; T2DM, diabetes mellitus type 2; HF, heart failure; BP, blood pressure.

The initiation of SGLT2is becomes relevant from Stage 2 onward. In Stage 2, CV risk reduction begins with lifestyle changes, followed by targeted pharmacological therapy to control BP, lipid levels, and blood glucose, and to prevent the onset of CKD. SGLT2is lower BP and provide both cardioprotective and nephroprotective effects in patients with T2DM. In Stage 3, early introduction of SGLT2is is advised to delay the progression to clinical HF and advanced CKD. By Stage 4, their use is strongly encouraged, supported by robust evidence demonstrating their effectiveness in lowering the risk of adverse CV and renal events. (Figure 2)

Taken together, we propose the following recommendations to guide the use of SGLT2is, alone or in combination with GLP-1 RAs or GIP/GLP-1 RAs, across the main components of CRM syndrome, informed by current guidelines and pivotal trials:

1. Obesity: SGLT2is provide modest weight loss when used alone. Greater reductions in absolute weight are achieved in combination with GLP-1 RAs. Given that SGLT2is and GLP-1 RAs are frequently co-administered for glycemic control and have a favorable safety profile, it is reasonable to consider adding an SGLT2i in obese patients with T2DM who are already on a GLP-1 RA.
2. Arterial Hypertension: SGLT2is modestly lower blood pressure and are not indicated as first-line therapy for hypertension. They may be included as part of antidiabetic treatment in patients with T2DM and hypertension.
3. High-risk T2DM: SGLT2is are indicated as first-line treatment.
4. HFpEF/HFrEF: SGLT2is are strongly recommended for HF across the ejection-fraction spectrum, regardless of diabetes status. Patients with T2DM, BMI ≥ 27 kg/m^2^, and HFpEF benefit from combination with GLP-1 RAs.
5. Acute HF: SGLT2is are recommended after clinical stabilization to enhance decongestion and improve clinical outcomes.
6. Acute MI: SGLT2is may be considered prior to and after PCI in high-risk STEMI patients. It may also reduce the risk of contrast-induced acute kidney injury.
7. ASCVD: SGLT2is are strongly recommended for patients with T2DM and ASCVD.
8. Diabetic CKD: SGLT2is are strongly recommended for patients with T2DM and eGFR ≥ 20 mL/min/1.73 m^2^.
9. NASH/NAFLD: SGLT2is improve hepatic steatosis. They may be reasonably added in patients with obesity who are already on GLP-1 RAs or GIP/GLP-1 RAs, particularly in cases of progressive liver disease.
10. Non-Diabetic Patients: SGLT2is demonstrate cardio-renal benefits in this population. Evidence is currently insufficient for these agents to provide clear guidance for obesity, arterial hypertension, or ASCVD. Regarding CKD, SGLT2is are indicated for eGFR ≥ 20 mL/min/1.73 m^2^ with UACR ≥ 200 mg/g (≥20 mg/mmol), or in those with HF regardless of albuminuria. For adults with eGFR 20–45 mL/min/1.73 m^2^ and UACR < 200 mg/g (<20 mg/mmol), SGLT2is may be considered as a treatment option.
11. Frail/Older Patients: SGLT2is should be continued when indicated, with enhanced monitoring for potential side effects.
12. OSA: Evidence for the efficacy of SGLT2is is limited, and their use cannot be routinely recommended [216].

Given the complexity of the CRM syndrome and the need for a comprehensive, holistic approach, the establishment of dedicated cardiometabolic clinics is imperative. Such initiatives are expected to improve patient outcomes and advance understanding of this multifaceted disease.

## 9. Conclusions

The emergence of SGLT2is represents a significant breakthrough in the treatment of CKM syndrome. Initially developed as antihyperglycemic agents, SGLT2is have consistently proven effects beyond glucose lowering, including renoprotective and cardioprotective properties, and improvements in lipid profile, hypertension, and obesity. Continued research is needed to better clarify the comprehensive impact of SGLT2is and to define their precise role in the treatment strategy for CRM syndrome. As findings from clinical trials and real-world data keep emerging, future guidelines will likely shift toward more individualized and holistic approaches to managing CRM syndrome, with SGLT2is positioned to be a key component.

## Figures and Tables

**Table 1:** Summary of Trials Evaluating SGLT2 Inhibitors Across the Components of Cardio-Renal-Metabolic Syndrome.

Trial/Author	SGLT2is	Primary Endpoint	Population	Follow-Up (Months)	Main Outcomes
HFrEF					
DAPA-HF [86]	Dapagliflozin	Composite of worsening HF (hospitalization or urgent visit requiring IV therapy) or CV death	4744 adults (≥18 years) with LVEF ≤ 40% and NYHA class II–IV symptoms	18	Dapagliflozin was associated with a reduction in the primary composite outcome (HR: 0.74; 95% CI: 0.65–0.85), total HHF (HR: 0.70; 95% CI: 0.59–0.83), and CV death (HR: 0.82; 95% CI: 0.69–0.98).
EMPEROR-Reduced [87]	Empagliflozin	Primary composite outcome of CV death or HHF (including first and recurrent events)	3730 adults (≥18 years) with LVEF ≤ 40% and NYHA class II–IV symptoms	16	Empagliflozin treatment resulted in a reduction in the primary composite outcome (HR: 0.75; 95% CI: 0.65–0.86) and a decreased risk of first HHF (HR: 0.69; 95% CI: 0.59–0.81), with no significant difference observed in CV death
HFpEF					
EMPEROR-Preserved [88]	Empagliflozin	Primary composite outcome of CV death or HHF (including first and recurrent events)	5988 adults (≥18 years) with LVEF > 40% and NYHA class II–IV symptoms	26	Empagliflozin reduced the risk of the primary composite outcome (HR: 0.79; 95% CI: 0.69–0.90) and total HHF (HR: 0.71; 95% CI: 0.60–0.83), with no significant effect observed on CV death (HR: 0.91; 95% CI: 0.76–1.09).
DELIVER [89]	Dapagliflozin	Composite of worsening HF (hospitalization or urgent visit requiring IV therapy) or CV death	10,584 adults (≥18 years) with LVEF > 40% and NYHA class II–IV symptoms, including those with improved LVEF	28	Dapagliflozin lowered the risk of the primary composite outcome (HR: 0.82; 95% CI: 0.73–0.92) and reduced the incidence of worsening HF events (HR: 0.79; 95% CI: 0.69–0.91), with no significant difference observed in CV death between groups (HR: 0.88; 95% CI: 0.74–1.05).
SOLOIST-WHF [90]	Sotagliflozin	Total number of CV deaths, hospitalizations, and urgent HF visits (first and recurrent events)	1222 adults (18–85 years) with T2DM hospitalized for worsening HF and treated with intravenous diuretics	9	Sotagliflozin significantly reduced primary endpoint events (HR: 0.67; 95% CI: 0.52–0.85)
CKD					
DAPA-CKD [91]	Dapagliflozin	Time-to-event analysis of first occurrence of ≥50% decline in eGFR, end-stage kidney disease (dialysis ≥ 28 days, transplant, or eGFR < 15 mL/min/1.73 m^2^ for ≥28 days), or death from renal or CV causes	4304 adults (≥18 years) with eGFR 25 to <75 mL/min/1.73 m^2^, UACR 200 to <5000 mg/g, and stable RAS inhibitor therapy for ≥4 weeks before randomization	29	Dapagliflozin reduced the risk of the primary composite outcome (HR: 0.61; 95% CI: 0.51–0.72), lowered the incidence of the composite of sustained ≥50% eGFR decline, end-stage kidney disease, or renal death (HR: 0.56; 95% CI: 0.45–0.68), decreased the risk of CV death or HHF (HR: 0.71; 95% CI: 0.55–0.92), and reduced all-cause mortality (HR: 0.69; 95% CI: 0.53–0.88).
EMPA-KIDNEY [92]	Empagliflozin	First occurrence of kidney disease progression (end-stage kidney disease defined as initiation of maintenance dialysis or kidney transplantation, sustained eGFR < 10 mL/min/1.73 m^2^, sustained ≥ 40% decline from baseline eGFR, or death due to renal causes) or CV death	6609 adults (≥18 years) with either eGFR ≥ 20 to <45 mL/min/1.73 m^2^ (regardless of UACR) or eGFR ≥ 45 to <90 mL/min/1.73 m^2^ with UACR ≥ 200 mg/g; on stable dose of a single RAS inhibitor	24	Empagliflozin lowered the risk of progression of kidney disease or CV death (HR: 0.72; 95% CI: 0.64–0.82) and reduced all-cause hospitalization (HR: 0.86; 95% CI: 0.78–0.95), while no significant differences were observed between groups in HHF, CV death (4.0% vs. 4.6%), or all-cause mortality (4.5% vs. 5.1%).
CREDENCE [93]	Canagliflozin	Composite outcome of end-stage kidney disease (dialysis ≥ 30 days, kidney transplant, or eGFR < 15 mL/min/1.73 m^2^ for ≥30 days), sustained doubling of serum creatinine from baseline (≥30 days), or death due to renal or cardiovascular causes	4401 adults (≥30 years) with T2DM, eGFR 30 to <90 mL/min/1.73 m^2^, UACR 300 to <5000 mg/g, and stable RAS inhibitor therapy for ≥4 weeks prior to randomization	31	Canagliflozin reduced the risk of the primary outcome by 30% compared to placebo (HR: 0.70; 95% CI: 0.59–0.82), lowered the renal-specific composite outcome by 34% (HR: 0.66; 95% CI: 0.53–0.81), decreased the risk of end-stage kidney disease by 32% (HR: 0.68; 95% CI: 0.54–0.86), reduced the risk of CV death, MI, or stroke (HR: 0.80; 95% CI: 0.67–0.95), and significantly lowered HHF (HR: 0.61; 95% CI: 0.47–0.80), with no significant differences observed in rates of amputation or fracture.
T2DM					
EMPA-REG OUTCOME [94]	Empagliflozin	Primary composite outcome: CV death, nonfatal MI, or nonfatal stroke	7020 adults (≥18 years) with T2DM at high risk for CV events	37	Empagliflozin reduced the primary composite outcome compared to placebo (10.5% vs. 12.1%; HR 0.86; 95% CI 0.74–0.99; *p* = 0.04). No significant differences were seen in MI or stroke rates. However, empagliflozin significantly lowered CV death (3.7% vs. 5.9%; 38% RRR), HHF (2.7% vs. 4.1%; 35% RRR), and all-cause mortality (5.7% vs. 8.3%; 32% RRR).
DECLARE–TIMI 58 [95]	Dapagliflozin	Primary composite outcome of CV death, nonfatal MI, or nonfatal stroke	17,160 adults (≥18 years) with T2DM who had or were at risk for ASCVD	50	Dapagliflozin demonstrated noninferiority for MACE (HR 0.93; 95% CI 0.84–1.03; *p* = 0.17) without a significant reduction in MACE rates. It significantly reduced CV death or HHF (HR 0.83; 95% CI 0.73–0.95; *p* = 0.005), driven by fewer HHF (HR 0.73; 95% CI 0.61–0.88). Renal events were also reduced (HR 0.76; 95% CI 0.67–0.87), with no significant difference in all-cause mortality (HR 0.93; 95% CI 0.82–1.04).
CANVAS Program [96]	Canagliflozin	Primary composite outcome of CV death, nonfatal MI, or nonfatal stroke	10,142 adults (≥18 years) with T2DM and an elevated risk of CVD	47	Canagliflozin lowered the primary outcome rate compared to placebo (26.9 vs. 31.5 events per 1000 patient-years; HR 0.86; 95% CI 0.75–0.97; *p* < 0.001 for noninferiority; *p* = 0.02 for superiority). While renal outcomes did not meet formal statistical significance per the prespecified testing sequence, canagliflozin showed potential benefits in slowing albuminuria progression (HR 0.73; 95% CI 0.67–0.79) and reducing a composite of sustained ≥40% eGFR decline, renal-replacement therapy, or renal death (HR 0.60; 95% CI 0.47–0.77).
Hypertension					
EMPA-REG BP [97]	Empagliflozin	Reduction in BP, tolerance, safety	825 adults with T2DM and hypertension	3	Empagliflozin significantly lowered blood pressure in patients with T2DM and hypertension, demonstrating good tolerability
Obesity					
Liu XY/Meta-analysis [54]	Empagliflozin, Dapagliflozin, Canagliflozin	Efficacy and safety. Evaluation of glucose lowering, BP, weight loss.	11,162 adults with T2DM	19	SGLT2is significantly reduced body weight (for 1-year result, WMD: −2.477; 95% CI: −2.568 to −2.385; I^2^ = 0.0%; for 2 years result, WMD: −2.990; 95% CI: −3.642 to −2.337; I^2^ = 0.0%)

Abbreviations: SGLT2is, sodium–glucose co-transporter-2 inhibitors; HFrEF, heart failure with reduced ejection fraction; HFpEF, heart failure with preserved ejection fraction; HF, heart failure; IV, intravenous; CV, cardiovascular; LVEF, left ventricular ejection fraction; NYHA, New York Heart Association; HR, hazard ratio; CI, confidence interval; HHF, hospitalization for heart failure; T2DM, type 2 diabetes mellitus; eGFR, estimated glomerular filtration rate; UACR, urinary albumin-creatinine ratio; MI, myocardial infarction; RAS, renin–angiotensin system; RRR, relative risk reduction; BP, blood pressure; WMD, weighted mean difference.

## Data Availability

No new data were created or analyzed in this study.
